# Hospital Expenditure

**Published:** 1902-11-01

**Authors:** 


### EDITOR'S LETTER-BOX.

[Our Correspondents are reminded that prolixity is a great bar to publication, and that brevity of style and conciseness of statement greatly facilitate early insertion.]

### HOSPITAL EXPENDITURE.

The Manager of Bovril, Limited, writes:—

Sir,—Our attention has been directed to a letter from a "Puzzled Matron," and to the reply thereto by the author of "Hospital Expenditure," in your issue of the 13th ult., touching *inter alia* on the cost of beef tea in hospitals.

May we be allowed, as a matter of interest to all concerned in hospital management, to point out that a large and increasing number of institutions have recognised the wisdom of availing themselves of the advances made in modern dietetics, by replacing beef tea by preparations far less costly and much preferable from a nutritive and stimulating standpoint.

All authorities are agreed in regarding as entirely erroneous the value popularly assigned to beef tea. It contains practically no nutritive matter, and possesses merely stimulating properties. On analytical grounds alone it is therefore not to be wondered at that the preparation with which we are identified should have been adopted by so many institutions.

More directly bearing, however, on the inquiry of your correspondent is the question of economy. The price of 4d. per lb., for beef, quoted by you from "The Hospital Commissariat," is much lower than that usually paid; but even at this contract rate the saving through using our preparation instead would amount annually to a large sum in most hospitals. Bovril costs less than 3d. per pint (at our special rates to hospitals), and requires practically no time or trouble in the making. Beef tea, on the other hand, involves several hours of constant supervision and much hand labour in the preparation, while the incidental outlay on fuel is very large. The expenditure under these headings may fairly be assumed as adding 50 per cent. to the cost. That is to say, the cost per pint of beef tea would ultimately amount to 6d.—more than double the price of our preparation.

We could furnish matrons with some striking figures supplied to us by the officials of various institutions—figures which bear out fully the above statement. In some the saving amounts to several hundred pounds annually since the adoption of Bovril.

